# Supplementary material for: Joule spectroscopy of hybrid superconductor–semiconductor nanodevices
Source: Nat Commun. 2023 May 19;14:2873. doi: 10.1038/s41467-023-38533-2 (PMC10199083; doi:10.1038/s41467-023-38533-2)
Supplement: Supplementary file 1 — Supplementary Information [file 41467_2023_38533_MOESM1_ESM.pdf]

Supplementary Information
Joule spectroscopy of hybrid superconductor-semiconductor nanodevices

A. Ibabe,[1,3,*] M. Gómez,[1,3,*] G. O. Steffensen,[2,3] T. Kanne,[4] J. Nygård,[4] A. Levy Yeyati,[2,3] and E. J. H. Lee[1,3,†]

[1]*Departamento de Física de la Materia Condensada, Universidad Autónoma de Madrid, Madrid, Spain*

[2]*Departamento de Física Teórica de la Materia Condensada, Universidad Autónoma de Madrid, Madrid, Spain*

[3]*Condensed Matter Physics Center (IFIMAC), Universidad Autónoma de Madrid, Madrid, Spain*

[4]*Center for Quantum Devices, Niels Bohr Institute, University of Copenhagen, Copenhagen, Denmark*

CONTENTS

S1. Extended data figures S3

S2. Supplementary experimental data S8

A. Properties of the epitaxial Al shell S8

B. Features related to the superconductivity of the Ti/Al contacts S9

C. Determining device parameters S12

D. Cooling power by electron-phonon coupling S15

S3. Transport theory S16

A. Pair-broken superconductor S16

B. Lead thermal balance S18

C. Schematic Theory for dips S20

D. Keldysh-Floquet transport theory S21

References S25

* These authors have contributed equally to this work.

† eduardo.lee@uam.es

S1. EXTENDED DATA FIGURES

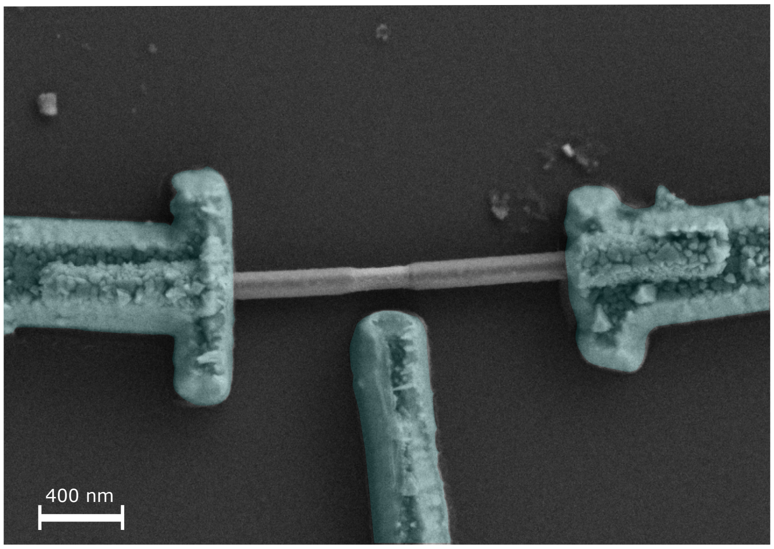

FIG. S1. | **Hybrid full-shell Al-InAs junctions**. Electron micrograph (false color) of a device that is lithographically similar to the devices studied in the main text.

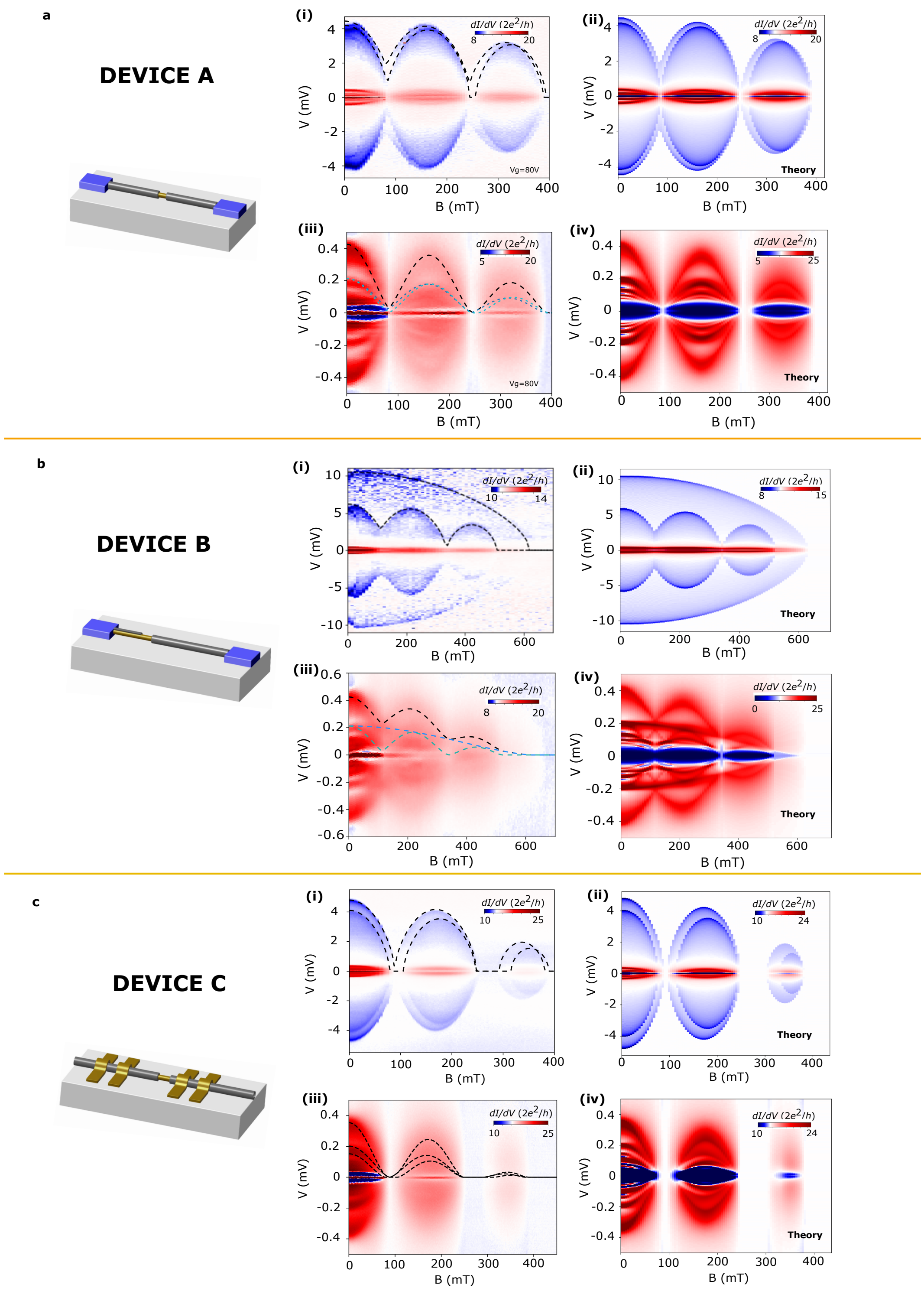

FIG. S2. | **Joule spectroscopy characterization of devices A, B and C**. For each device we plot: (i) the Joule spectrum of the leads and (ii) dI/dV at low-V as a function of B, and Floquet-Keldysh calculations of the (iii) high-V and (iv) low-V transport response.

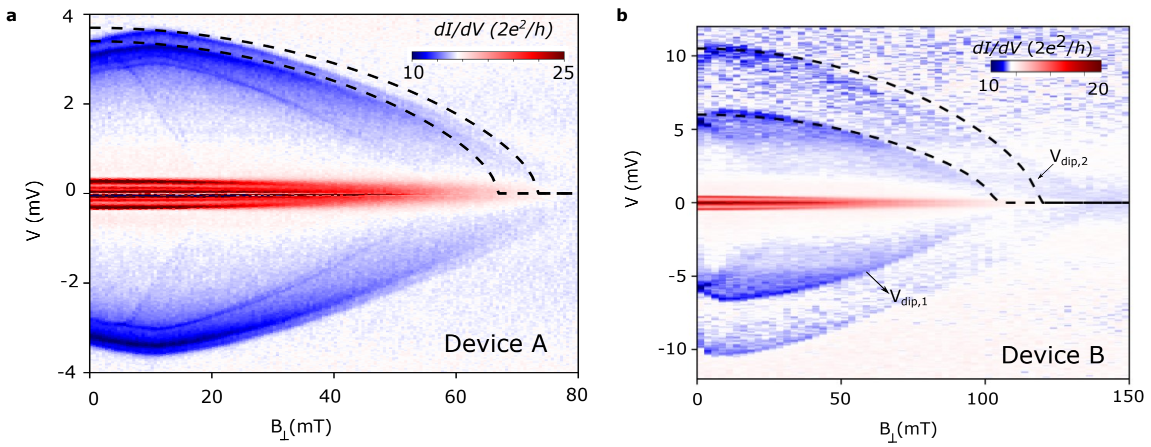

FIG. S3. | **Perpendicular magnetic field dependences.** $dI/dV(V)$ as a function of perpendicular magnetic field, $B_\perp$ for devices A (panel **a**) and B (panel **b**). Dashed lines show predictions of the AG theory using the same parameters obtained from fitting the data with the nearly parallel magnetic field, B.

.

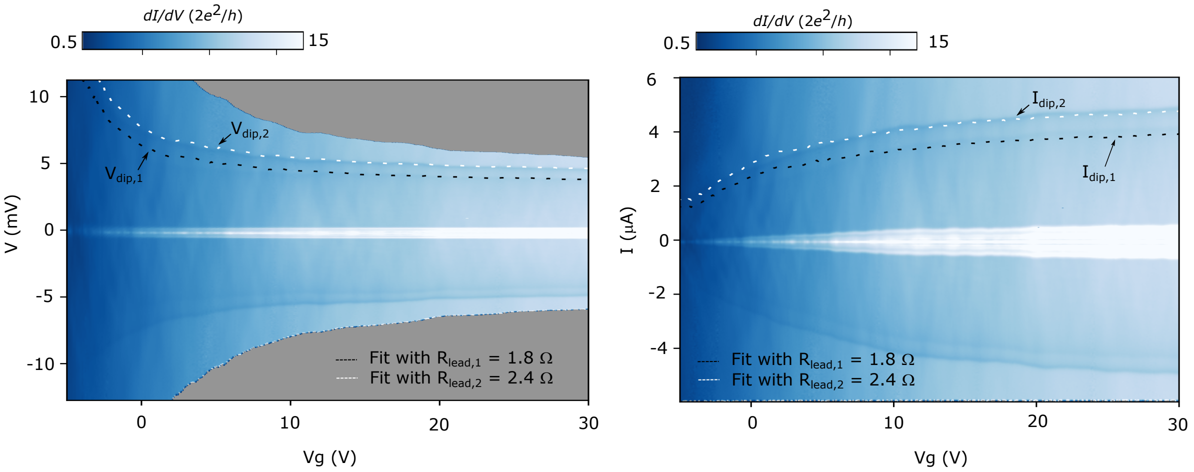

FIG. S4. | **Gate dependence of the dip features in device C**. $dI/dV(V)$ (left panel) and $dI/dV(I)$ (right panel) measured in a 4-terminal configuration as a function of the gate voltage. Notice that no post-processing of the data is required to obtain the real voltage drop across the device (see Methods), as the measurement is not affected by series resistances in the experimental setup (e.g., the resistance of the cryogenic filters). The dashed lines are fits to Eq. (3) in the main text with a single free fitting parameter per dip, $R_{lead,1}$ and $R_{lead,2}$. An excellent agreement is obtained between the experimental data and the fits, from which we obtain $R_{lead,1} \approx 1.8\ \Omega$ and $R_{lead,2} \approx 2.4\ \Omega$. Note that for this analysis, we have used the two different superconducting critical temperatures of the leads, namely $T_{c,1} = 0.98$ K and $T_{c,2} = 1.31$ K, which result from the inverse superconducting proximity effect.

S2. SUPPLEMENTARY EXPERIMENTAL DATA

A. Properties of the epitaxial Al shell

We present here a characterization of the epitaxial Al shell of nanowires from the same batch as that used for devices A, B and C. We have fabricated devices with a 4-terminal geometry and with angle-evaporated Cr(2.5 nm)/Au(80 nm) contacts, similar to device C. In this case, however, the Al shell was not etched. Current-biased measurements were taken at low temperatures and with an external magnetic field, B. Such a characterization was aimed at estimating relevant parameters of the Al shell, such as the normal state resistance, R_n, the superconducting coherence length, ξ_S, and the critical current, I_c^{shell}, to compare with the results obtained from our analyses of the dips in the main text.

Fig. S5 displays a typical $dV/dI(I, B)$ measurement, where I is the current bias. Note that the measurements were taken by sweeping I from negative to positive values and, as such, features in the negative/retrapping branch may be affected by heating effects. We will not discuss this in further detail, as it is outside of the scope of this work. By measuring a total of 5 devices, we have observed a distribution of critical current, $I_c^{shell} \approx 10 - 25\mu$A (taken at positive I). These values are at least 2-3 times larger than the highest values measured for I_{dip}, reinforcing that the reported dips are not related to the critical current of the shell.

Concerning the normal state resistance of the shell, we define $R_n = dV/dI(I > I_c^{shell})$. In Fig. S5, we plot R_n as a function of the distance between the voltage probes, L. By applying a linear fit to the datapoints, we estimate $R_n/L \approx 11$ Ω/m. As mentioned in the main text, the R_{lead} values obtained by fitting the dips agree very well with this estimate.

We now evaluate the superconducting coherence length of the epitaxial shell. We estimated ξ_S from R_n by applying the methodology described in ref. [1]. In brief, in the dirty limit of superconductors, the coherence length is given by $\xi_\mathrm{S} = \sqrt{\pi\hbar v_\mathrm{F} l_e / 24 k_B T_c(B=0)}$, where $v_\mathrm{F} = 2.03 \times 10^6$ m/s is the electron Fermi velocity in Al, and l_e is the mean free path. This latter parameter is obtained from the resistivity of the Al shell. By taking R_n, and considering the geometrical dimensions of the shell in each of our devices, we estimate $l_e \sim 2$ nm. From this value, we calculate ξ_S for the 5 measured nanowires, obtaining a distribution in the range of 75-105 nm, consistent with the values obtained from the AG fitting of the

dips in the main text.

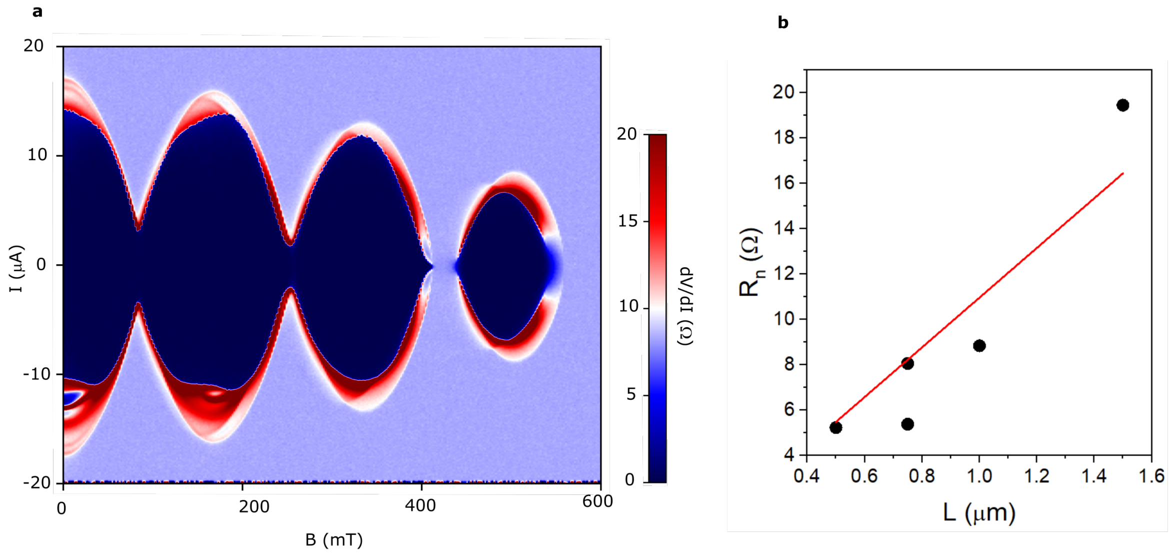

FIG. S5. **Characterization of the epitaxial Al shell**. **a**, $dV/dI(I)$ measurement taken as a function of B. **b**, Length dependence of the normal state resistance of the Al shell. L corresponds to the distance between the voltage probes of the device.

B. Features related to the superconductivity of the Ti/Al contacts

In this section, we will discuss dip-related features that are observed in all devices with superconducting lithographic contacts (Ti/Al), but that are absent when the contacts are normal (Cr/Au).

We start by addressing the faint dI/dV dips that were mentioned in passing in the discussion of Fig. 2b (labeled as $V_{dip,lith}$). These dips are more prominently seen in measurements taken as a function of T or B. Indeed, they are also present in the B-field dependences in Figs. 3a and 4b, although their visibility is compromised by the lower resolution of those measurements. We show in Fig. S6a a higher resolution $dI/dV(V, B)$ measurement for device A, focusing on lower magnetic fields. This measurement is similar to Fig. 3a, but it was taken in a different cool-down. For this reason, we note that even though both measurements were taken at the same gate voltage ($V_g = 80$ V), R_J (and consequently $V_{dip,i}$) are slightly different owing to a small shift in the pinch-off voltage of the device upon thermal

cycling. Interestingly, $R_{lead,i}$ remains unchanged for the different cool-downs, reinforcing that it is a property of the leads and not of the junction. Importantly, we note that the behavior of the faint dips is consistent with the superconductivity of 240 nm-thick Al films with lateral dimensions $\sim \mu$m. Notably, their critical temperature ($T_{c,lith}(B = 0) \approx .1.1$ K) is lower than that of the epitaxial shell ($T_{c,i}(B = 0) \approx 1.35$ K), and their critical magnetic field is $\sim 20 - 50$ mT. We thus conclude that the faint dips indeed have their origin in the lithographic Ti/Al contacts. We do not discuss these dips further, as they do not affect the main conclusions of this work.

We also attribute the slight increase of $V_{dip,i}$ at low fields (up to $\sim$ 20 mT) to the Ti/Al contacts. As we mentioned in the main text, this effect leads to a small discrepancy between the data and the AG fitting. Fig. S6 clearly demonstrates that the dips in devices with Cr/Au contacts do not show such a discrepancy at low B. In analogy to the previous effect, we speculate that the present behavior is also related to the superconductor-to-normal transition of the Ti/Al film. In brief, we believe that the closing of the superconducting gap of the Ti/Al contacts slightly improves the thermal transport from the junction to the bath, leading to a small renormalization of $R_{lead,i}$. Indeed, we estimate that $R_{lead,i}$ at $B = 0$ is approx. 10% higher than at $B = 20$ mT, suggesting a slightly higher thermal resistance.

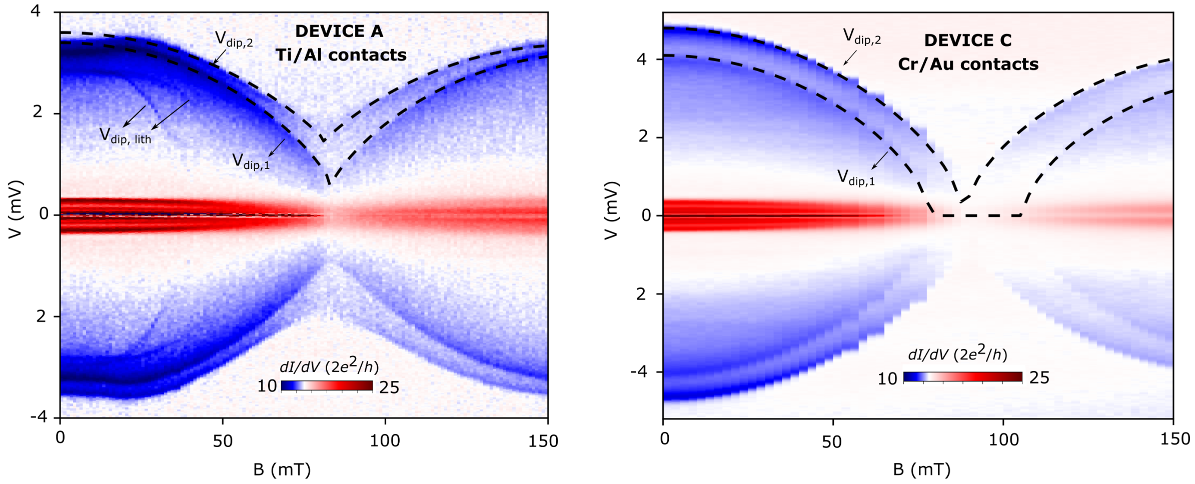

FIG. S6. **Dip features in devices with superconducting (left panel) and normal (right panel) lithographic contacts**. Devices with Ti/Al contacts show additional faint dips that are suppressed for low magnetic fields. They also show a slight increase in $V_{dip,i}$ upon applying B from zero to ~ 20 mT.

C. Determining device parameters

In this section we provide detail on the fitting of parameters for device A, B and C. From the main text it is already established how zero-field critical temperature, $T_c(B=0)$, and lead resistance, R_{lead}, are obtained by monitoring dips under change of cryostat temperature, T_{bath}, and junction gate, V_g, respectively. Additionally, for a given V_g we measure the zero-field normal resistance, R_J, and maximal excess current, $\max(I_{exc,1}(V)+I_{exc,2})(V)$, which we use to fit the number of transmission channels, N, and the transmission of each channel, τ, as to produce the same ratio of excess current to resistance in theory calculations. Realistically, each channel, j, will have a different transmission and fitting each τ_j can be achieved by precise fitting of MAR peaks [2]. As we primarily focus on high-bias measurements, and to keep the number of fitting parameters low, we deem this procedure not worthwhile.

Next, we elaborate on the fitting of the Little-Parks lobes observed in $V_{dip}(B)$ as a function of field, and compare it to expected wire parameters. Little-Parks oscillations of $T_c(B)/T_c(0) \approx V_{dip}(B)/V_{dip}(0)$ in a superconducting thin cylinder in the dirty limit, is described by [3, 4],

$$\ln\left(\frac{T_c(\alpha)}{T_c(0)}\right) = \Psi\left(\frac{1}{2}\right) - \Psi\left(\frac{1}{2} + \frac{\alpha}{2\pi k_B T_c(\alpha)}\right), \tag{1}$$

where Ψ is the digamma function, and α the pair-breaking parameter. As a perfect mechanical alignment between the nanowire axis and the applied magnetic field is not experimentally feasible we leave a small angle, θ, as an additional fitting parameter, resulting in a parallel and a perpendicular contribution to the magnetic field: $B_\parallel = B\cos\theta$, $B_\perp = B\sin\theta$. Difference in θ between two leads we attribute to a possible curvature of the nanowire. Consequently, the total pair-breaking is given by $\alpha = \alpha_\parallel + \alpha_\perp$ [1, 5] with,

$$\alpha_\parallel = \frac{4{\xi_S}^2 T_c(0)}{A}\left[\left(n - \frac{\Phi_\parallel}{\Phi_0}\right)^2 + \frac{t_S^2}{d^2}\left(\frac{\Phi_\parallel^2}{\Phi_0^2} + \frac{n^2}{3}\right)\right], \tag{2}$$

$$\alpha_\perp = \frac{4{\xi_S}^2 T_c(0)\lambda}{A}\frac{\Phi_\perp^2}{\Phi_0^2}. \tag{3}$$

Here n denotes the fluxoid quantum number, $\Phi_\parallel = B_\parallel A$, $\Phi_\perp = B_\perp A$, $A = \pi d^2/4$, and λ is a free fitting parameter determining the perpendicular contribution to pair-breaking. For the purpose of fitting this function is characterized by the following four components,

$$B_p = \frac{\Phi_0}{A\cos\theta}, \quad C_1 = \frac{4\xi_S^2 T_c(0)}{A}, \quad C_2 = \frac{1}{3}\frac{t_S^2}{\pi A} + \lambda\frac{\sin^2\theta}{\cos^2\theta}, \quad C_3 = \lambda A^2, \tag{4}$$

where B_p is the measured LP periodicity, C_1 sets the amplitude of periodic oscillations, C_2 the decay at integer flux, $\Phi_{\|}/\Phi_0 = n$, and C_3 the decay for a perpendicular field ($\theta \approx \pi/2$). A given measurement of V_{dip} as a function of parallel magnetic field, possibly with a small θ, in combination with a perpendicular field measurement with $\theta \approx \pi/2$, can be fitted by the components $\{B_p, C_1, C_2, C_3\}$, and consequently any parameters yielding identical $\{B_p, C_1, C_2, C_3\}$ also provides a fit. Here we assume perfect alignment in the perpendicular direction as a small parallel component is negligible, while a small perpendicular component to a parallel alignment is not. If we assume that $\{A, t_S, \xi_S, \lambda, \theta\}$ are all free parameters a unique fit cannot be obtained. Nonetheless, the space of possible fits for dips 1&2 in device A and dip 2 in device B is restricted to $\theta \in \{0°, 10°\}$ as shell thickness, t_S, otherwise becomes complex in order to keep C_2 constant.

By fixing $t_S = 15$ nm (from fabrication $t_S \approx 20$ nm) and $\lambda = 1.7$ a unique fit is obtained for all dips, with corresponding values shown in tables below. The resulting fits for all devices can be seen in Extended Data Fig. 1-2. For this choice, we find from B_p that d_A, $d_C \approx 125$ nm and $d_B \approx 105$ nm (with A, B and C indicating device) comparable to the nominal length of 135 nm from fabrication. In the allowed range of freedom for θ, parameters $\{A, \xi_S, \lambda\}$ only varies within third digit precision, consequently we can conclude that the coherence length, ξ_S, must be different between lead 1 and 2 in order to obtain a good fit. This highlights the ability of Joule spectroscopy to extract the coherence lengths of each lead independently. Device B lead 2 is a special case as no Little-Parks oscillation is observed, and we concluded that the Al shell is not doubly connected. As a function of $B_{\|}$ a monotone decaying trend of $V_{dip,2}$ is observed which is fitted by setting $\alpha_{\|} = 0$ and fitting θ. Consequently, the angle, θ, for dip 2 in device B should only be understood as a fitting parameter since we lack knowledge of the state of the Al shell.

Device A

lead	T_{bath} [K]	N	τ	R_{lead} [Ω]	$T_c(0)$ [K]	ξ_S [nm]	B_p [mT]	λ	t_S [nm]	θ [deg]
1	0.25	16	0.675	4.4	1.35(1.4)	100	166	1.7	15	3.7
2	0.25	16	0.675	3.8	1.35(1.4)	90	162	1.7	15	5.6

TABLE S1. **Parameters of Device A**. T_{bath}, N and τ are all tuneable, and values shown here correspond to those in Fig. 3. The $T_c(0)$ value in parentheses is the one used in theory calculations. Other quantities are given by: $\Delta_i(0) = 1.76 k_B T_{c,i}(0)$, $R_J = 1/G_0 N\tau$, and $A = \Phi_0/B_p \cos\theta$.

Device B

lead	T_{bath} [K]	N	τ	R_{lead} [Ω]	$T_c(0)$ [K]	ξ_S [nm]	B_p [mT]	λ	t_S [nm]	θ [deg]
1	0.01	15	0.69	2.0	1.35(1.4)	75	225	1.7	15	7
2	0.01	15	0.69	0.7	1.35(1.4)	65	225	1.7	15	11

TABLE S2. **Parameters of Device B**. T_{bath}, N and τ are all tuneable, and values shown here correspond to those in Fig. 4. The $T_c(0)$ value in parentheses is the one used in theory calculations. Other quantities are given by: $\Delta_i(0) = 1.76 k_B T_{c,i}(0)$, $R_J = 1/G_0 N\tau$, and $A = \Phi_0/B_p \cos\theta$. Note that for lead 2 we put $\alpha_{\parallel}(B) = 0$ and B_p is fitted to yield the correct perpendicular decay for $\lambda = 1.7$. The angle, θ, should only be regarded as a fitting parameter for lead 2.

Device C

lead	T_{bath} [K]	N	τ	R_{lead} [Ω]	$T_c(0)$ [K]	ξ_S [nm]	B_p [mT]	λ	t_S [nm]	θ [deg]
1	0.25	21	0.64	1.8	1.0(1.0)	115	182	1.7	15	5.8
2	0.25	21	0.64	2.4(2.7)	1.33(1.4)	100	176	1.7	15	7.7

TABLE S3. **Parameters of Device C**. T_{bath}, N and τ are all tuneable, and values shown here correspond to those in Fig. 4. The $T_c(0)$ and R_{lead} value in parentheses is the one used in theory calculations. Difference in R_{lead} stems from difference in $T_c(0)$. Other quantities are given by: $\Delta_i(0) = 1.76 k_B T_{c,i}(0)$, $R_J = 1/G_0 N\tau$, and $A = \Phi_0/B_p \cos\theta$.

D. Cooling power by electron-phonon coupling

We estimate here the cooling power provided by electron-phonon coupling in the epitaxial Al shell, $P_{e\text{-}ph}$, to support our assumption that, in our devices, cooling predominantly occurs via quasiparticles in the leads. Following refs. [6, 7], we write the heat balance equation:

$$P_{e\text{-}ph} = \Sigma U (T_{el}^5 - T_{ph}^5), \tag{5}$$

where $\Sigma = 1.8$ nW/μm^3 K^5 is the Al electron-phonon coupling parameter [8], $U \approx 7.07 \times 10^{-3} \mu\text{m}^3$ is the volume of the Al shell (assuming a NW core diameter of 135 nm, a shell thickness of 15 nm, and a length of 1 μm), T_{el} is the electron temperature, and T_{ph} is the phonon temperature, which we take to be equal to T_{bath}. At the superconductor-to-normal metal transition of the leads, the electron temperature reaches the superconducting critical temperature, $T_c = 1.35$ K. By assuming $T_{ph} = 0.25$ K, we obtain $P_{e\text{-}ph} \sim 0.057$ nW, which is more than two orders of magnitude lower than the measured $P_{dip,i} \sim 10$ nW. We therefore conclude that heat diffusion by quasiparticles in the leads is a more efficient cooling mechanism in our devices.

S3. TRANSPORT THEORY

In this section we elaborate on the main theoretical results relating the measurements of high voltage conductance dips with properties of the junction and leads. Simple approximate relations connecting the conductance dips with lead and junction parameters, such as T_c, are derived by assuming that thermal transport is solely mediated by lead quasi-particles, and that for a given power input each lead independently reaches thermal equilibrium. Finally to validate these relations we self-consistently calculate the power each lead receives from joule heating through the use of Keldysh-Floquet transport methodology, accounting for both pair-breaking, asymmetric leads, and Andreev reflection to all orders. Results from this approach are compared to experimental data both in the supplement and in the main text.

A. Pair-broken superconductor

The application of either a parallel or perpendicular magnetic field induces a pair-breaking, α, in the leads, and because of the small mean-free path compared to coherence length the resulting pair-broken superconductivity can be described by Abrikosov-Gor'kov theory [3, 4, 9]. In this subsection we iterate the key components of this theory used in our calculations. Under the influence of pair-breaking, the quasi-classical retarded Green function is given by

$$g^R(\omega) = -i\pi\nu_F \frac{u(\omega) - \tau_x}{\sqrt{u(\omega)^2 - 1}}, \tag{6}$$

where ν_F is the density of state at the Fermi level and τ_x a pauli matrix in Nambu space. The complex number $u(\omega)$ is obtained as the solution of

$$u(\omega)\Delta(\alpha, T) = \omega + i\alpha \frac{u(\omega)}{\sqrt{(u(\omega)^2 - 1)}}. \tag{7}$$

For a given $\Delta(\alpha, T)$ this equation can be expressed as a fourth order polynomial with root $u(\omega)$ chosen as to satisfy appropriate boundary conditions of the Green function. For the pairing parameter self-consistency with the Green function demands,

$$\Delta(\alpha, T) = \nu_F U \int_0^{\hbar\omega_D} d\omega \, \mathrm{Re} \frac{1}{\sqrt{u(\omega)^2 - 1}} \tanh \frac{1}{2} \frac{\omega}{k_B T}, \tag{8}$$

where U is the strength of the interaction, assumed weak, T denotes temperature, and ω_D the Debye frequency. The various scales appearing in this problem are connected by standard

BCS relations; $\Delta_0 = 2\hbar\omega_D e^{-1/\nu_F U}$ and $k_B T_{c0} = \frac{2e^\gamma}{\pi}\hbar\omega_D e^{-1/\nu_F U}$ with $T_{c0} = T_c(\alpha = 0)$, $\Delta_0 = \Delta(\alpha = 0, T = 0)$ and γ denoting Euler's constant. For finite pair-breaking and zero-temperature a closed form solution of $\Delta(\alpha, 0)$ exist,

$$\ln \frac{\Delta_0}{\Delta(\alpha,0)} = \begin{cases} -\frac{\pi}{4}\frac{\alpha}{\Delta(\alpha,0)} & \text{if } \alpha \leq \Delta(\alpha,0), \\ -\ln \frac{\alpha+\sqrt{\alpha^2-\Delta(\alpha,0)^2}}{\Delta(\alpha,0)} + \frac{\sqrt{\alpha^2-\Delta(\alpha,0)^2}}{2\alpha} - \frac{1}{2}\arctan \frac{\Delta(\alpha,0)}{\sqrt{\alpha^2-\Delta(\alpha,0)^2}} & \text{if } \alpha \geq \Delta(\alpha,0), \end{cases} \tag{9}$$

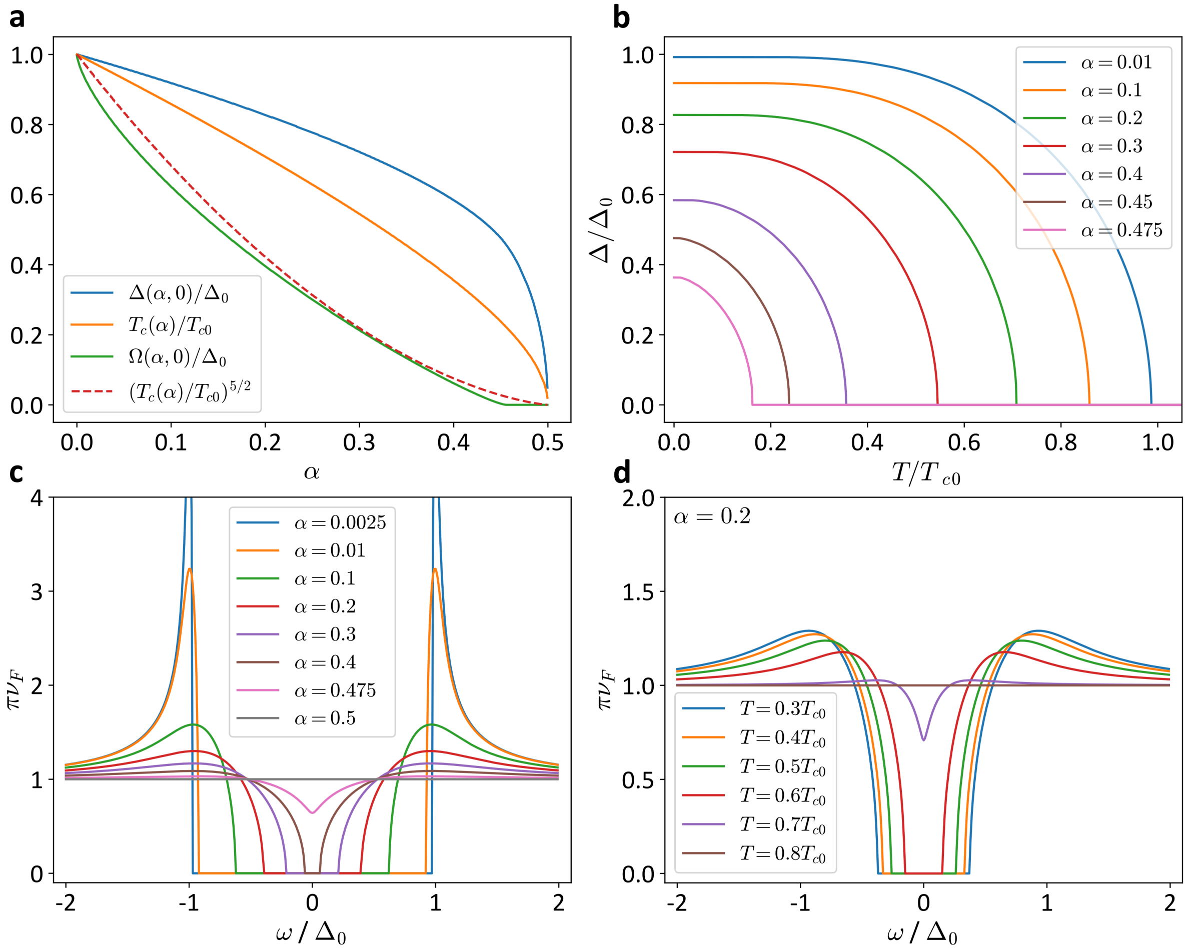

FIG. S7. **Effects of pair-breaking**. **a** Pairing parameter $\Delta(\alpha, 0)$, critical temperature $T_c(\alpha)$, and the spectral gap $\Omega(\alpha, 0)$ as a function of pairing. **b** Numerical solutions of eq. (10) for $\Delta(\alpha, T)$ for various α. **c** Spectral function $A(\omega) = -\text{Im } g^R_{11}(\omega)$ at zero temperature. **d** The effect of temperature on the spectral function for finite pair-breaking. In all plots α is in units of Δ_0.

which can be solved by intersect. In the case of finite temperature eq. (8) has to be solved

as an integral equation, and using BCS relations we express it as,

$$\frac{2\Delta(\alpha,T)}{\Delta_0}\log N = \int_0^N dx\,\mathrm{Re}\frac{1}{\sqrt{u(\Delta_0 x/2)^2-1}}\tanh\frac{\Delta_0 x}{4T}, \tag{10}$$

where N is a numerical parameter chosen sufficiently large number as to assure the integrand approaches $\frac{2\Delta(\alpha,T)}{\Delta_0 x}$ for $x \to N$. For a given α and T eq. (7) and eq. (10) can be jointly solved numerically to obtain $\Delta(\alpha,T)$ and $u(\omega)$, with the size of N determining precision. The above relations allow evaluation of the retarded Green function, eq. (6), for any value of α and T from which the spectral function $A(\omega) = -\mathrm{Im}\, g_{11}^R(\omega)$ can be obtained. One characteristic of a pair-broken supercondcutor is that the spectral gap, denoted $\Omega(\alpha,T)$, is not equal to the pairing parameter, $\Delta(\alpha,T)$, as in the case of BCS superconductivity but instead given by,

$$\Omega(\alpha,T) = \left(\Delta(\alpha,T)^{\frac{2}{3}} - \alpha^{\frac{2}{3}}\right)^{\frac{3}{2}}. \tag{11}$$

In Fig. S7 we show various quantities characterizing superconductivity dependence on pair-breaking and temperature. In Fig. S7a an approximate relation relating spectral gap to critical temperature, $\Omega(\alpha,0)/\Delta_0 \approx (T_c(\alpha)/T_{c0})^{5/2}$, is additionally shown.

B. Lead thermal balance

As a consequence of electron tunneling across the junction a non-equilibrium distribution of high energy quasi-particles emerge on the left and right lead. In the following we assume that on a given lead this distribution relaxes to an equilibrium distribution releasing a power P at the lead interface. We further assume that all heat diffusion through the epitaxial aluminium stems from activated quasi-particles and solve for thermal equilibrium. This derivation largely follows calculations of *Tomi et al.* [10], here expanded to also include pair-breaking.

We model the epitaxial aluminium leads as a 1D wire of length L and cross sectional area S. Thermal equilibrium requires that the power passing through each segment of wire be equal, such that a lead temperature distribution, $T(x)$, stabilizes. This condition amounts to the heat diffusion equation,

$$S\kappa_S(\alpha,T)\frac{dT}{dx} = -P, \tag{12}$$

with the thermal conductivity, $\kappa_S(\alpha,T)$, given by the analogous Wiedemann-Franz law for

a pair-broken superconductor [11],

$$\kappa_S(\alpha, T) = \frac{4k_B^2\sigma}{e^2}T\int_{\frac{\Omega(\alpha,T)}{2k_BT}}^{\infty} dx\ \frac{x^2}{\cosh^2 x}h(2k_BTx, \alpha, T) \tag{13}$$

where the effect of pair-breaking is encapsulated in the function,

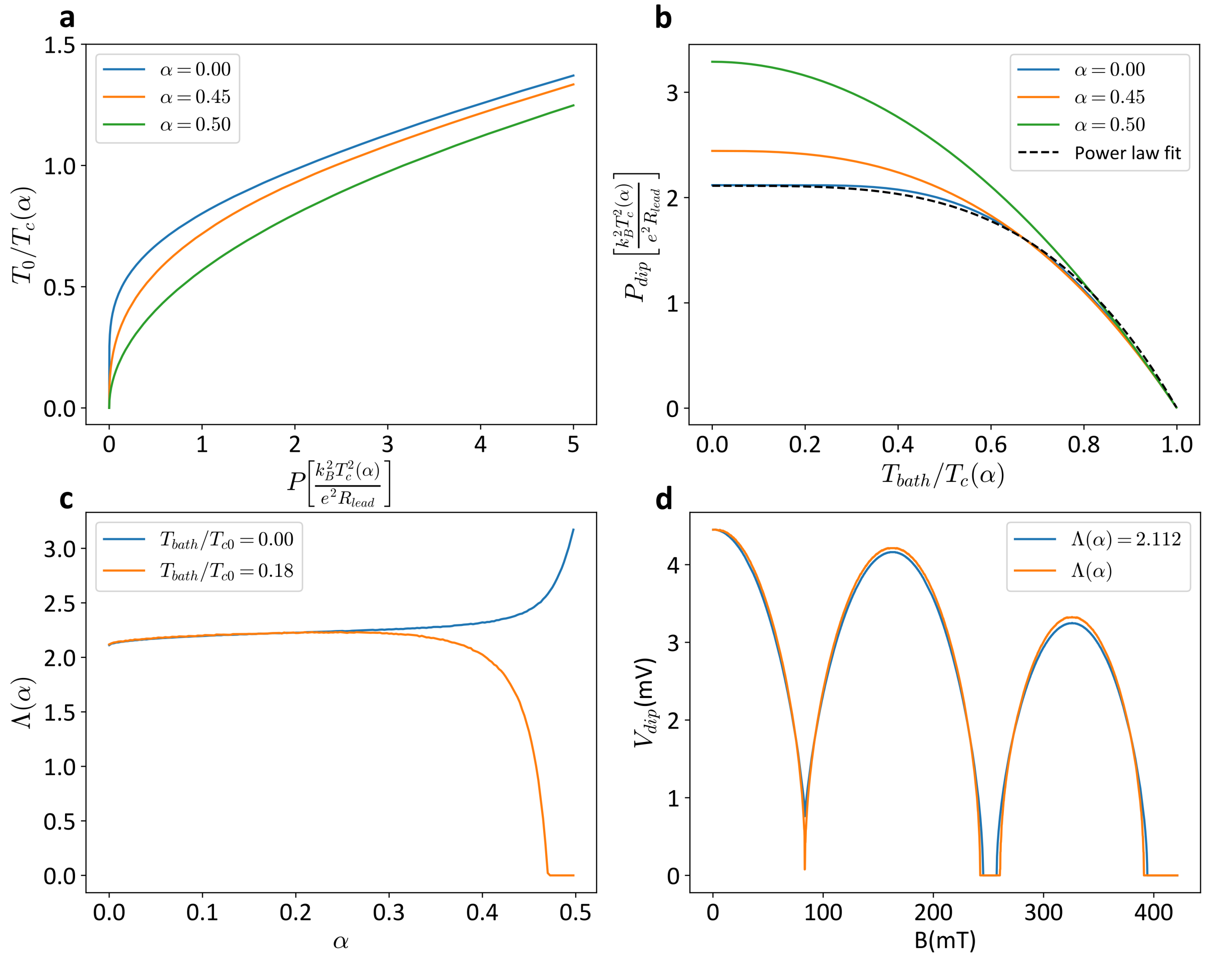

FIG. S8. **Solutions to quasi-particle heat diffusion**. **a** Interface temperature as a function of injected power from eq. (15). **b** Scaling of dip power, P_{dip}, with bath temperature, T_{bath}, obtained from eq. (16). **c** $\Lambda(\alpha, T_{bath})$ as a function of pair-breaking for zero temperature and $T_{bath} = 0.18T_{c0}$ corresponding to $T_{bath} = 250$ mK and $T_{c0} = 1.4$ K. **d** Expected position of $V_{dip,1}$ (obtained from eq. (21) using device A parameters, see table S1) calculated with $\Lambda(\alpha, T_{bath})$ assumed constant and by using eq. (17). α is in units of Δ_0.

$$h(\omega, \alpha, T) = \left(\mathrm{Re}\frac{u(\omega)}{\sqrt{u(\omega)^2 - 1}}\right)^2 - \left(\mathrm{Re}\frac{1}{\sqrt{u(\omega)^2 - 1}}\right)^2, \tag{14}$$

$h(\omega, \alpha, T)$ depends on α and T through $u(\omega)$'s dependence of $\Delta(\alpha, T)$ in eq. (7). Integrating eq. (12) across the length of the wire and imposing the boundary condition $T(x = L) = T_{bath}$ and $T(x = 0) = T_0$, with T_{bath} denoting environment temperature and T_0 temperature at the junction interface, yields,

$$P = \frac{8k_B^2}{e^2 R_{lead}} \int_{T_{bath}}^{T_0} dT\, T \int_{\frac{\Omega(\alpha,T)}{2k_B T}}^{\infty} dx\, \frac{x^2}{\cosh^2 x} h(2k_B T x, \alpha, T), \tag{15}$$

with lead resistance defined as $R_{lead} = 2L/\sigma S$. A conductance dip occurs whenever $T_0 = T_c(\alpha)$ and the required power can be expressed as,

$$P_{dip} = \Lambda(\alpha, T_{bath}) \frac{k_B^2 T_c^2(\alpha)}{e^2 R_{lead}} \tag{16}$$

with the thermal properties of the leads described by the unitless function,

$$\Lambda(\alpha, T_{bath}) = \frac{8}{T_c^2(\alpha)} \int_{T_{bath}}^{T_c(\alpha)} dT\, T \int_{\frac{\Omega(\alpha,T)}{2k_B T}}^{\infty} dx\, \frac{x^2}{\cosh^2 x} h(2k_B T x, \alpha, T). \tag{17}$$

This function is bounded by $\pi^2/3 \geq \Lambda(\alpha, T) \geq 0.0$, with the lower bound reached for $T_{bath} \geq T_c(\alpha)$ when no additional power is required to drive the interface normal, and the upper bound reached for $\alpha/\Delta_0 = 0.5$ when the lead becomes metallic and most thermally conductive. In the zero temperature BCS limit $\Lambda(0, 0) = 2.112$ [10] and remains approximately constant so far $T_c(\alpha) \geq T_{bath}$, as shown in Fig S8c-d. For $\alpha = 0$ one obtains the approximate power law,

$$\Lambda(0, T_{bath}) \approx 2.112 \left(1 - \frac{T_{bath}^{3.6}}{T_{c0}^{3.6}}\right), \tag{18}$$

which is compared to the exact curve in Fig. S8b. The fitted power, 3.6, attempts to bridge the transition from an initial exponentially suppressed curve for $T_{bath} \ll T_{c0}$ to a second order closing, T_{bath}^2/T_{c0}^2, at $T_{bath} \approx T_{c0}$ [10].

C. Schematic Theory for dips

Next, we present a schematic calculation to obtain the bias position of the dips. In the high bias regime, $eV \gg \Delta_1 + \Delta_2$ with $\Delta_i = \Delta_i(\alpha_i = 0, T_{bath} = 0)$ for lead 1 and 2, the excess current can be described as originating from two independent S - N junctions with the total current across the junction given by,

$$I = \frac{V}{R_J} + I_{exs,1}(\Delta_1, \alpha_1, T_{0,1}) + I_{exs,2}(\Delta_2, \alpha_2, T_{0,2}), \tag{19}$$

where excess current, $I_{exs,i}$, depends non-trivially on both temperature and pair-breaking. The power deposited on either lead is given by,

$$P_{1(2)} = \frac{V^2}{2R_J} + VI_{exs,2(1)}(\Delta_{2(1)}, \alpha_{2(1)}, T_{0,2(1)}). \tag{20}$$

To obtain interface temperature $T_{0,i}$ exactly requires a self-consistent treatment; for a given P_i one finds $T_{0,i}$ from eq. (15), but a change of $T_{0,i}$ modifies P_i. If the normal contribution to current greatly exceeds the excess current at a thermal dip, $V_{dip,i}/R_J \gg I_{exs,1},\ I_{exs,2}$, this self-consistency is negligible as $P_1 \approx P_2 \approx V^2_{dip,i}/2R_J$ yielding,

$$V_{dip,i} = R_J I_{dip,i} = \sqrt{2\Lambda(\alpha_i, T_{bath})}\sqrt{\frac{R_J}{R_{lead,i}}}\frac{k_B T_{c,i}(\alpha_i)}{e}, \tag{21}$$

identical to eq. (3) of the main text. Under the application of a magnetic field both $\Lambda(\alpha_i, T_{bath})$ and $T_{c,i}(\alpha)$ are simultaneously modified, but as $\Lambda(\alpha_i, T_{bath})$ can be approximated as a constant (see Fig. S8d) changes of $V_{dip,i}$ directly correspond to changes of $T_{c,i}$. These equations constitute the main results enabling Joule spectroscopy.

D. Keldysh-Floquet transport theory

In this subsection we use the Keldysh-Floquet Green function technique for a pair-broken superconductor [12] to self-consistently in $T_{0,i}$ calculate DC current, I, plotted in theory figures of the main text. These calculations additionally support that previous assumptions of constant $\Lambda(\alpha_i, T_{bath})$ and $V_{dip,i}/R_J \gg I_{exs,i}$ is reasonable, and allow us to compare low-bias MAR structure with high bias dips. We consider transport to occur between the left and right Al superconducting shell, which are described by quasi-classical Green functions, and model the junction as a generic contact with N transmission eigenvalues τ_i of the corresponding normal-state scattering matrix. Using appropriate boundary conditions for the quasi-classical Greens functions, transport can be described via the matrix current, [13, 14],

$$\check{I}(t) = \frac{e^2}{h}\sum_n \frac{\tau_n\,[\check{g}_1, \check{g}_2]_-}{1 - \frac{1}{2}\tau_n + \frac{1}{4}\tau_n\,[\check{g}_1, \check{g}_2]_+}(t,t) \tag{22}$$

where $-(+)$ describe (anti-)commutators and with time-convolution assumed in the matrix structure, $\check{g}_1\check{g}_2(t,t') = \int_{-\infty}^{\infty} dt''\check{g}_1(t,t'')\check{g}_2(t'',t')$. The Green functions are written in Nambu-

Keldysh space,

$$\check{g}_i = \begin{pmatrix} \bar{g}_i^R & \bar{g}_i^K \\ 0 & \bar{g}_i^A \end{pmatrix}, \quad \bar{g}_1(t,t') = \frac{\tau_z}{i\pi\nu_{F,1}} g_1(t-t'), \quad \bar{g}_2(t,t') = \frac{\tau_z}{i\pi\nu_{F,2}} e^{ieVt\tau_z/\hbar} g_2(t-t') e^{-ieVt'\tau_z/\hbar} \tag{23}$$

where $g_i^R(t-t') = \int_{-\infty}^{\infty} d\omega\, g_i^R(\omega) e^{-i\omega(t-t')}$ and $g_i^R(\omega)$ is given by eq. (6) with $g_i^A(\omega) = \left[g_i^R(\omega)\right]^\dagger$ and $g_i^K(\omega) = \left(g_i^R(\omega) - g_i^A(\omega)\right) \tanh\left(\omega/2T_{0,i}\right)$. In this framework the Green functions of lead i are completely specified by parameters $\{\Delta_i, \nu_{F,i}, \alpha_i, T_{0,i}\}$. The gauge part, $e^{ieVt\tau_z/\hbar}$, originates from the AC Josephson effect where an applied DC voltage drop creates explicit time-dependence, and where τ_z denotes a pauli matrix in Nambu space. To highlight the connection between the quasi-classical and tunneling descriptions we rewrite the matrix current using Dyson series,

$$\check{I}(t) = \frac{4e^2}{h} \sum_n \frac{\tau_n}{4-2\tau_n} \left[\check{g}_1, \check{g}_2\right]_- \check{M}_{+n} = \frac{4e^2}{h} \sum_n b_n \left(\check{g}_2\check{g}_1\check{M}_{21,n} - \check{g}_1\check{g}_2\check{M}_{12,n}\right) \tag{24}$$

with $\tau_n = 4b_n^2/(1+b_n)^2$ and,

$$\check{M}_{+n} = 1 - \frac{\tau_n}{4-2\tau_n} \left[\check{g}_1, \check{g}_2\right]_+ \check{M}_{+n}, \quad \text{and} \quad \check{M}_{ij,n} = 1 + b_n \check{g}_i \check{g}_j \check{M}_{ij,n}. \tag{25}$$

These expression are obtained by utilizing the following identity $\check{g}_i\check{g}_i = I$ and we recognize $b_n = \pi^2 \nu_{F,1}\nu_{F,2}|t_n|^2$ where t_n describes the tunneling amplitude in a corresponding tunneling model. Lastly we identify $b_n\check{g}_2\check{g}_1\check{M}_{21,n} = \sqrt{b_n}\check{G}_{21,n}$ with the dressed Green functions defined via typical equation-of-motion structure,

$$\check{G}_{21,n} = \check{g}_2\sqrt{b_n}\check{G}_{11,n} \quad \text{and} \quad \check{G}_{11,n} = \check{g}_1 + \check{g}_1\sqrt{b_n}\check{g}_2\sqrt{b_n}\check{G}_{11,n}, \tag{26}$$

such that the matrix current is given by,

$$\check{I}(t) = \frac{4e^2}{h} \sum_n \left(\sqrt{b_n}\check{G}_{21}(t,t) - \sqrt{b_n}\check{G}_{12}(t,t)\right), \tag{27}$$

identical to equations obtained from S - S tunneling models [15]. From the matrix current we obtain the charge and energy current [14],

$$\begin{aligned} I(t) &= \frac{1}{4e} \mathrm{Tr}\, \tau_z \check{I}^K(t), \\ P_1(t) &= \frac{1}{8e} \mathrm{Tr}\left[\epsilon \check{I}^K(t) + \check{I}^K(t)\epsilon\right], \\ P_2(t) &= I(t)V - P_L(t), \end{aligned} \tag{28}$$

with $\check{I}^K(t)$ indicating the Keldysh component of the matrix current and $\epsilon(t,t') = i\partial_t\delta(t-t')$. Assuming that the system reach a time-periodic non-equilibrium steady state, $\check{g}_i(t,t') = \check{g}_i(t+T, t'+T)$ with $T = 2\pi\hbar/eV$, we can transform time-convolutions into Floquet matrix structure. Considering only the DC component, corresponding to the zeroth Floquet band, we obtain the following equations for the currents,

$$
\begin{aligned}
I &= \frac{2e}{h}\sum_n \int_{-\infty}^{\infty} d\omega \mathrm{Re}\ \mathrm{Tr}\left[\tau_z\, b_n \underline{\check{M}}^R_{21,n}\left(\underline{\check{g}}^R_2 \underline{\check{g}}^<_1 + \underline{\check{g}}^<_2 \underline{\check{g}}^A_1\right)\underline{\check{M}}^A_{21,n}\right]_{00}, \\
P_1 &= \frac{2e}{h}\sum_n \int_{-\infty}^{\infty} d\omega \mathrm{Re}\ \mathrm{Tr}\left[\omega\, b_n \underline{\check{M}}^R_{21,n}\left(\underline{\bar{g}}^R_2 \underline{\bar{g}}^<_1 + \underline{\bar{g}}^<_2 \underline{\bar{g}}^A_1\right)\underline{\check{M}}^A_{21,n}\right]_{00}, \\
P_2 &= IV - P_1,
\end{aligned}
\tag{29}
$$

with the 'underline' indicating Floquet matrix structure and 00 indicating initial and final Floquet index. Entrances in Floquet matrices are given by,

$$
\bar{g}^X_{i,nm}(\omega) = \frac{1}{T}\int_0^T dt\, e^{i(n-m)eVt/\hbar}\int_{-\infty}^{\infty} dt'\, e^{i(\omega+meV/\hbar)(t-t')}\bar{g}^X_i(t,t'), \tag{30}
$$

with $X \in \{R, L, <\}$ and $\bar{g}^<_{i,nm}(\omega) = \left(\bar{g}^A_{i,nm}(\omega) - \bar{g}^R_{i,nm}(\omega)\right) n_F(\omega + meV/\hbar, T_{0,i})$. In this framework the product $\underline{\bar{g}}_1\underline{\bar{g}}_2$ forms a block tridiagonal matrix in Nambu-Floquet space, which $\underline{\check{M}}_{21,n}$ is a convergent series of. Consesquently for a given b_n the number of included Floquet bands can be truncated to obtain I and P_i to any given precision. The numerical results presented in the main paper are obtained in the following way; For a given magnetic field we obtain α_i from Little-Park theory which together with an initial guess of $T_{0,i}$ yields $\Delta_i(\alpha_i, T_{0,i})$ and $g^X_i(t,t)$ via eq. (10) and eq. (6). For a given bias, eV, we then calculate I and P_i using eq. (29) including sufficient Floquent bands as to assure convergence. From P_i we update $T_{i,0}$ using eq. (15), which is used to update $\Delta_i(\alpha, T_{0,i})$ and $g^X_i(t,t')$ and recalculate I and P_i until convergence of $T_{0,i}$ is achieved. This procedure assures that thermal transport across the junction stemming from asymmetry in leads and heat diffusion is properly accounted for in a self-consistent manner.

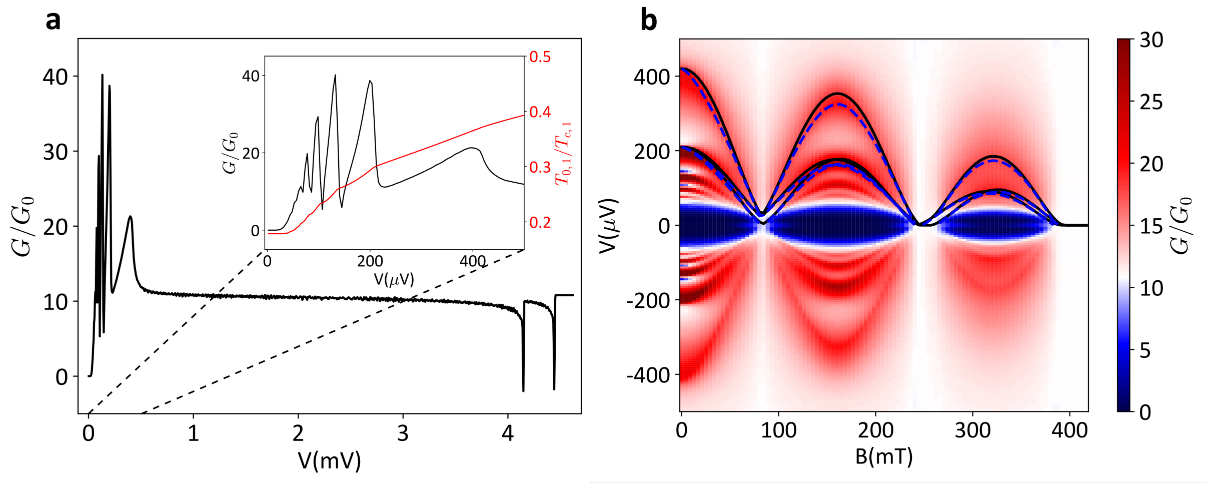

FIG. S9. **Self-consistent calculation of transport**. **a** High resolution conductance line-cut at zero magnetic field for device A. Inset shows low-bias MAR structure and $T_{0,1}$ obtained self-consistently. **b** Low-bias conductance map showing the effect of magnetic field on MAR structure. Black lines and dashed blue lines indicate expected position of MAR resonances obtained from $\Omega_i(\alpha_i, 0)$ and eq. (31) respectively. Plots are made using device A parameters, see table S1.

Results of self-consistent Floquet Keldysh calculations are shown both in the supplement and main text, and by using experimentally extracted parameters (see tables in subsection S2 C) we find a good agreement between experiment and theory. A full comparison for all devices can seen in Extended Data Figure 1. Simulations shown in both Extended Data and in the main text are performed with a finite coarse-graining set to approximately match experimental resolution. Using finer graining we find that both low-bias MAR features and high-bias conductance dips contain narrow peaks not fully resolved in experiment, as shown in Fig. S9a which is identical to Fig. 1d of the main text except graining. For a BCS superconductor with no pair-breaking MAR steps for odd n appear at bias $V = (\Delta_1 + \Delta_2)/en$, and for even n at $V = \Delta_i/en$. For finite pair-breaking, $\alpha_i \neq 0$, we find that MAR steps instead appear as fractions of the spectral gap, $\Omega_i(\alpha_i, T_{0,i})$, in a similar manner. In experiment, however, spectral gaps are not directly extractable from measurements of high bias dips, but as shown in Fig. S8a for zero temperature one approximately finds $\frac{\Omega_i(\alpha_i,0)}{\Delta_i} \approx \left(\frac{T_{c,i}(\alpha_i)}{T_{c,i}(0)}\right)^{5/2}$ yielding MAR steps at,

$$V = \begin{cases} \frac{\Delta_1}{en}\left(\frac{T_{c,1}(\alpha_1)}{T_{c,1}(0)}\right)^{5/2} + \frac{\Delta_2}{en}\left(\frac{T_{c,2}(\alpha_2)}{T_{c,2}(0)}\right)^{5/2} & \text{if } n \text{ is odd,} \\ \frac{\Delta_i}{en}\left(\frac{T_{c,i}(\alpha_i)}{T_{c,i}(0)}\right)^{5/2} & \text{if } n \text{ is even.} \end{cases} \quad (31)$$

Approximating $\Lambda(\alpha, T_{bath})$ as constant renders $T_{c,i}(\alpha_i)$ proportional to $V_{dip,i}$ and consequently $T_{c,i}(\alpha)/T_{c,i}(0) = V_{dip,i}(\alpha)/V_{dip,i}(0)$. This last relation allows one to fit low-bias MAR structure directly from measurements of high-bias conductance dips. In Fig. S9b we show a simulation of low-bias conductance for device A alongside fits of MAR lines yielding good agreement between conductance peaks and MAR integers.

Lastly, it should be noted that the above analysis does not account for the low bias supercurrent branch around $V \approx 0$. Consequently the zero-bias conductance peak observed in experiment is not reproduced by numerical simulations.

---

[1] S. Vaitiekėnas, P. Krogstrup, and C. M. Marcus, Anomalous metallic phase in tunable destructive superconductors, Phys. Rev. B **101**, 060507 (2020).

[2] M. F. Goffman, C. Urbina, H. Pothier, J. Nygård, C. M. Marcus, and P. Krogstrup, Conduction channels of an InAs-Al nanowire Josephson weak link, New J. Phys. **19**, 092002 (2017).

[3] A. A. Abrikosov and L. P. Gor'kov, Contribution to the theory of superconducting alloys with paramagnetic impurities, Zh. Eksp. Teor. Fiz **39**, 1781 (1960).

[4] S. Skalski, O. Betbeder-Matibet, and P. R. Weiss, Properties of Superconducting Alloys Containing Paramagnetic Impurities, Phys. Rev. **136**, A1500 (1964).

[5] A. Vekris, J. C. Estrada Saldaña, J. de Bruijckere, S. Lorić, T. Kanne, M. Marnauza, D. Olsteins, J. Nygård, and K. Grove-Rasmussen, Asymmetric Little–Parks oscillations in full shell double nanowires, Sci. Rep. **11**, 1 (2021).

[6] F. C. Wellstood, C. Urbina, and J. Clarke, Hot-electron effects in metals, Phys. Rev. B **49**, 5942 (1994).

[7] H. Courtois, M. Meschke, J. T. Peltonen, and J. P. Pekola, Origin of hysteresis in a proximity Josephson junction, Phys. Rev. Lett. **101**, 067002 (2008).

[8] V. F. Maisi, S. V. Lotkhov, A. Kemppinen, A. Heimes, J. T. Muhonen, and J. P. Pekola, Excitation of single quasiparticles in a small superconducting al island connected to normal-metal leads by tunnel junctions, Phys. Rev. Lett. **111**, 147001 (2013).

[9] I. Larkin, A., Superconductor of small dimensions in a strong magnetic field, Sov. Phys. JETP **21**, 153 (1965).

[10] M. Tomi, M. R. Samatov, A. S. Vasenko, A. Laitinen, P. Hakonen, and D. S. Golubev, Joule

heating effects in high-transparency Josephson junctions, Phys. Rev. B **104**, 134513 (2021).

[11] V. Ambegaokar and A. Griffin, Theory of the Thermal Conductivity of Superconducting Alloys with Paramagnetic Impurities, Phys. Rev. **137**, A1151 (1965).

[12] A. V. Zaitsev and D. V. Averin, Theory of ac Josephson Effect in Superconducting Constrictions, Phys. Rev. Lett. **80**, 3602 (1998).

[13] Y. V. Nazarov, Novel circuit theory of Andreev reflection, Superlattices Microstruct. **25**, 1221 (1999).

[14] P. Virtanen and F. Giazotto, Thermal transport through ac-driven transparent Josephson weak links, Phys. Rev. B **90**, 014511 (2014).

[15] J. C. Cuevas, A. Martín-Rodero, and A. L. Yeyati, Hamiltonian approach to the transport properties of superconducting quantum point contacts, Phys. Rev. B **54**, 7366 (1996).
